# Enrichment differentiation of human induced pluripotent stem cells into sinoatrial node-like cells by combined modulation of BMP, FGF, and RA signaling pathways

**DOI:** 10.1186/s13287-020-01794-5

**Published:** 2020-07-16

**Authors:** Feng Liu, Yibing Fang, Xiaojie Hou, Ying Yan, Haiying Xiao, Dongchuan Zuo, Jing Wen, Linli Wang, Zhichao Zhou, Xitong Dang, Rui Zhou, Bin Liao

**Affiliations:** 1 Department of Cardiac Macrovascular Surgery, Affiliated Hospital of Southwest Medical University, 3-319 Zhongshan Road, Luzhou, 646000 Sichuan China; 2 College of Integrated Traditional Chinese and Western Medicine, Southwest Medical University, Luzhou, 646000 Sichuan China; 3 The Key Laboratory of Medical Electrophysiology of Ministry of Education and Medical Electrophysiological Key Laboratory of Sichuan Province, Collaborative Innovation Center for Prevention and Treatment of Cardiovascular Disease of Sichuan Province, Institute of Cardiovascular Research, Southwest Medical University, 3-319 Zhongshan Road, Luzhou, 646000 Sichuan China; 4 Guangzhou Biocare Institute of Cancer, Guangzhou, 510663 Guangdong China; 5 Division of Cardiology, Department of Medicine, Karolinska University Hospital, Karolinska Institute, Stockholm, Sweden

**Keywords:** Human induced pluripotent stem cell (hiPSC), Sinus atrial node-like cell (SANLC), Bone morphogenetic protein (BMP) signaling, Fibroblast growth factor (FGF) signaling, Retinoic acid (RA) signaling

## Abstract

**Background:**

Biological pacemakers derived from pluripotent stem cell (PSC) have been considered as a potential therapeutic surrogate for sick sinus syndrome. So it is essential to develop highly efficient strategies for enrichment of sinoatrial node-like cells (SANLCs) as seed cells for biological pacemakers. It has been reported that BMP, FGF, and RA signaling pathways are involved in specification of different cardiomyocyte subtypes, pacemaker, ventricular, and atrial cells. We aimed to investigate whether combined modulation of BMP, FGF, and RA signaling pathways could enrich the differentiation of SANLC from human pluripotent stem cell (hiPSC).

**Methods:**

During the differentiation process from human induced pluripotent stem cell to cardiomyocyte through small molecule-based temporal modulation of the Wnt signaling pathway, signaling of BMP, FGF, and RA was manipulated at cardiac mesoderm stage. qRT-PCR, immunofluorescence, flow cytometry, and whole cell patch clamp were used to identify the SANLC.

**Results:**

qRT-PCR results showed that manipulating each one of bone morphogenetic protein (BMP), fibroblast growth factor (FGF), and retinoid acid (RA) signaling was effective for the upregulation of SANLC markers. Moreover, combined modulation of these three pathways displayed the best efficiency for the expression of SANLC markers, which was further confirmed at protein level using immunofluorescence and flow cytometry. Finally, the electrophysiological characteristics of upregulated SANLC were verified by patch clamp method.

**Conclusion:**

An efficient transgene-independent differentiation protocol for generating SANLC from hiPSC was developed, in which combined modulating BMP, FGF, and RA signaling at cardiac mesoderm stage generates SANLC at high efficiency. This may serve as a potential approach for biological pacemaker construction.

## Introduction

“Sick sinus syndrome” (SSS) is a group of heart arrhythmia caused by perturbed function of the sinus node that is composed of cardiac pacemaker cells. They include pathological and/or symptomatic sinus bradycardia, sinoatrial (SA) block, and tachycardia-bradycardia syndrome. Although implantation of electronic pacemaker has been one of the most effective treatments at present, it is associated with significant risks of infection, hemorrhage, and lead dislodging. Moreover, limited battery life and lack of autonomic neurohumoral responses further limit its usage [1, 2]. Therefore, the “biological pacemaker” derived from human pluripotent stem cells (hPSCs) may provide a promising alternative treatment.

Cardiomyocyte differentiation from hPSC is recognized as a powerful model to simulate the human embryonic heart development in vivo and is a promising source of cardiomyocytes for regenerative medicine. The embryonic heart development experiences several spatial-temporal stages from primitive streak to cardiac crescent and then to primitive heart tube, and the latter forms distinct anterior and posterior poles containing different mesodermal progenitors that give rise to different cardiomyocyte subtypes including ventricular, atrial, and sinoatrial node (SAN)-like cardiomyocytes (SANLCs) [3–5]. To date, most protocols were designed to generate heterogenous ventricular cardiomyocytes with a very small percentage of atrial and SANLC [6, 7]. Analyses of early developmental stages revealed that ventricular, atrial, and SAN-like cardiomyocytes were derived from different mesoderm cell populations that could be distinguished based on their expression of CD235a [8, 9], RALDH2 [8], and TBX18 [10–12], respectively. In addition, it was found that different signaling pathways were involved in different cardiomyocyte subtype specifications. For example, retinoic acid (RA) signaling at the mesoderm stage is required for atrial specification [8], while ventricular specification is highly dependent on the fibroblast growth factor (FGF) signaling [13–16]. A recent study showed that bone morphogenetic protein (BMP) signaling plays an important role in the specification of mesoderm progenitors into SANLC [17, 18]. These early discoveries suggest that hiPSC-induced cardiomyocytes can be directed to differentiate into SAN-like cardiomyocytes at cardiac mesoderm stage by activation of the BMP signaling pathways leading to SAN-like, and by simultaneous inhibition of signaling pathways leading to ventricular and atrial cardiomyocytes.

Consequently, we aimed to figure out a new working model of enriched differentiation of SANLC from hiPSC. We have established a developmental biology-based approach to enrich SANLC from hiPSC. We found in the present study that activation of BMP and simultaneous inhibition of RA and FGF signaling pathways during cardiac mesoderm stage of hiPSC differentiation can significantly enrich SAN-like cardiomyocytes. These findings will facilitate the study of human SAN development and may provide a rich source of cells for the development of biological pacemaker.

## Materials and methods

### Materials

The information of purchased reagents and cell line used in the study is as follows: LHpb-YaabC3 hiPSC (HNF-P30-P11, OSINGLAY BIO, China), BJ human foreskin fibroblast cell line (CRL2522, ATCC, USA), HN4 human embryonic stem cell (hESC) line (HES-P20-P9, OSINGLAY BIO, China), Cell culture media RPMI/1640 (11875093, Thermo Fisher Scientific, USA), DMEM/F12 media (11320082, Thermo Fisher Scientific, USA), medium for hiPSC BioCISO (BC-PM0001, OSINGLAY BIO, China), GSK3 inhibitor CHIR99021 (S1263, Sigma, USA), FGF inhibitor PD (3044, Tocris Bioscience, UK), Wnt inhibitor IWP2 (3533, Tocris Bioscience, UK), ROCK inhibitor Y27632 (1254, Tocris Bioscience, UK), BMP activator BMP4 (120-05ET, PEPROTECH, USA), RA inhibitor BMS (SML1149, Sigma, USA), B-27 supplement with (17504-044, Thermo Fisher Scientific, USA) or without insulin, Matrigel (354277, Corning, UK), TRIzol Reagent (15596026, Thermo Fisher Scientific, USA), and Real-time PCR reagents (208056, Qiagen, Germany). All primers/oligos were synthesized by Shenggong Biotech, China, and listed in Table 1. All other reagents, unless specified otherwise, were products of Sigma.

**Table 1:** Primer sets for qRT-PCR analysis

Gene	Direction	Sequence (5′–3′)	Product size (bp)
NANOG	Forward	TTTGTGGGCCTGAAGAAAACT	91
	Reverse	AGGGCTGTCCTGAATAAGCAG	
SOX2	Forward	GCCGAGTGGAAACTTTTGTCG	145
	Reverse	GGCAGCGTGTACTTATCCTTCT	
OCT4	Forward	CTGGGTTGATCCTCGGACCT	121
	Reverse	CCATCGGAGTTGCTCTCCA	
BRACHYURY	Forward	CAGTGGCAGTCTCAGGTTAAGAAGGA	122
	Reverse	CGCTACTGCAGGTGTGAGCAA	
MESP1	Forward	AGCCCAAGTGACAAGGGACAACT	82
	Reverse	AAGGAACCACTTCGAAGGTGCTGA	
NKX2-5	Forward	CAAGTGTGCGTCTGCCTTT	190
	Reverse	CAGCTCTTTCTTTTCGGCTCTA	
TNNT2	Forward	TTCACCAAAGATCTGCTCCTCGCT	111
	Reverse	TTATTACTGGTGTGGAGTGGGTGTGG	
SHOX2	Forward	CAAAGAGGATGCGAAAGGGAT	122
	Reverse	AGTGGGTCTCGTCAAAAAGCC	
TBX18	Forward	GACGATCTTTCTCCCATCAAGC	124
	Reverse	CTATCTTCAGGCGAGTAATCTGC	
TBX3	Forward	CCCGGTTCCACATTGTAAGAG	104
	Reverse	GTATGCAGTCACAGCGATGAAT	
HCN4	Forward	TGGACACCGCTATCAAAGTGG	157
	Reverse	CTGCCGAACATCCTTAGGGA	
MYL7	Forward	AAGCCATCCTGAGTGCCTTC	127
	Reverse	AACATCTGCTCCACCTCAGC	
MYL2	Forward	TGAGAGACACCTTTGCTGCC	139
	Reverse	GGGTCCGCTCCCTTAAGTTT	
MSX2	Forward	CTGGTGAAGCCCTTCGAGAC	133
	Reverse	ATATGTCCTCCTACTCCTGCCC	
TBX2	Forward	TACGCTTGTACGAGGAGCAC	157
	Reverse	CACGACTTCTCCTCAGCTCG	
ISL1	Forward	TCCCTATGTGTTGGTTGCGG	194
	Reverse	CATTTGATCCCGTACAACCTGA	
Cx30.2	Forward	CCGAGAACCTTGCCTTGGTA	98
	Reverse	GACAGAAACCGCTGACTCCA	
Cx43	Forward	TACCAAACAGCAGCGGAGTT	139
	Reverse	TGGGCACCACTCTTTTGCTT	
Cx40	Forward	AGAGTGTGAAGAAGCCCACG	70
	Reverse	AACAGATGCCAAAACTTCTGCT	
HCN1	Forward	GCCATGCTGAGCAAGTTGAG	178
	Reverse	TCAGCAGGCAAATCTCTCCA	
KCND2	Forward	GGGTCTTCGGCTAGCAAGTT	91
	Reverse	GCACCATGTCACCATACCCTA	
KCNK2	Forward	TGGAACAAGACTCCTTGCTGG	150
	Reverse	CTGCCGAACATCCTTAGGGA	
KCNN4	Forward	CCGAGAGGCAGGCTGTTAAT	81
	Reverse	AGCCGATGGTCAGGAATGTG	
KCNJ5	Forward	CCCACAACAGGGAGAGGTTC	170
	Reverse	AGCCATAGCTGGGATGTTGTT	
KCNQ1	Forward	GGGCCGCGTCTACAACTTC	169
	Reverse	CAGCACGATCTCCATCCAGAA	
CACNA1A	Forward	GTCTGGGGAAGAAGTGTCCG	151
	Reverse	GCTCCTCCCTTGGCAATCTT	
CACNB1	Forward	CCAGTGCCAAACAGAAGCAG	181
	Reverse	CGAGTGATGGAGATCCTGCC	
CACNA1C	Forward	GACGTGCTGTACTGGGTCAA	125
	Reverse	AACTCTCCGCTAAGCACACC	
SCN5A	Forward	GGAGGAGTCCAGCAAGCAG	194
	Reverse	AACTGTCCTCTGGGGTCTCA	
SCN3B	Forward	ACGCATTCTGTAGCCCAGAC	156
	Reverse	CTTCCAAGGCTCTCGCCTC	

### Cardiomyocyte differentiation from hiPSC

Cardiomyocyte differentiation was performed in a growth factor and serum-free system by temporal modulation of the canonical Wnt signaling pathway with GSK3 inhibitor (Gi) and Wnt inhibitor (Wi), known as the GiWi protocol [19]. Briefly, 80–90% confluent hiPSC was harvested using 0.5 mM EDTA and resuspended with hiPSC-maintaining medium at 0.5 × 10^5^ cells per milliliter. Two milliliters of the cell suspension was seeded per well in a 12-well Matrigel-coated plate with 2 ml of hiPSC medium at minus day 4. At day 0, the medium was refreshed with RPMI/B-27 containing CHIR99021 (10 μM, GSK3 inhibitor) without insulin and continued to incubate for 24 h. The medium was replaced with RPMI/B-27 without insulin for another 48 h. On day 3 of the differentiation (72 h after addition of CHIR99021), the medium was refreshed with RPMI/B-27 containing IWP2 (5 μM, Wnt inhibitor) without insulin for 48 h, followed by RPMI/B-27 without insulin from day 5 to 7. From day 7, the medium was refreshed with RPMI/B-27 containing insulin every 3 days. The beating cardiomyocytes can be seen as early as on day 8.

### Enriched differentiation of SAN-like cardiomyocytes from hiPSC by BMP4 (B), PD (P), and BMS (M) treatment

Based on the GiWi protocol that it caused highly efficient pan-cardiomyocytes as shown above, different concentrations of BMP4 (0, 1.25, 2.5, 5 ng/ml), PD (0, 480, 720, 960 nM), and BMS (0, 1 μM) were added alone between day 5 and 7 after differentiation for 2 days. The qRT-PCR analysis was performed to evaluate the mRNA levels of SAN markers at day 16 to determine the optimal concentration of each compound. To investigate the synergistical effect, the cells were treated with combinations of BMP4, PD, and BMS with the optimal concentration from day 5 to 7 during the differentiation process. The markers of pacemaker cells were evaluated by analysis of qRT-PCR, immunofluorescence, and flow cytometry at day 21. Electrophysiological characteristics were analyzed using action potential (AP) recording at day 60. The schematic protocol is shown in Fig. 4a.

### RNA isolation and qPCR

Total RNA was isolated using TRIzol method (15596026, Invitrogen, USA). One microgram of total RNA was reversely transcribed in a total volume of 10 μl with ReverTra Ace qPCR RT Master Mix kit (FSQ-201, TOYOBO, Japan) following the manufacturer’s instructions. The cDNA was diluted 3 times, and 1 μl was used for real-time PCR in a 20-μl reaction using SYBR Green Real Time PCR Mix (204143, Qiagen, Germany). The PCR conditions were 95 °C for 2 min, followed by 40 cycles of 95 °C for 20″ and 60 °C for 15″. All primers are listed in Table 1. The expression of target gene was normalized to that of GAPDH and calculated using the 2^-∆∆Ct^ method.

### Immunofluorescence

Single hiPSC cells and induced cardiomyocytes were seeded in a μ-Slide 8 well (80827, ibidi) coated with Matrigel at the density of 2 × 10^4^ per well for 48 h. Cells were fixed with 4% (w/v) paraformaldehyde (PFA) for 15 min at room temperature, permeabilized, blocked in 5% (w/v) BSA in PBS for 30 min and then incubated with the following primary antibodies: anti-OCT4 antibody (#2750, Cell Signaling Technology, USA), anti-NANOG antibody (#3580, Cell Signaling Technology, USA), anti-SSEA4 antibody (#4755, Cell Signaling Technology, USA), anti-TRA-1-60 antibody (#4746, Cell Signaling Technology, USA), anti-Ki67 antibody (ab15580, abcam, USA), anti-NKX2.5 antibody (ab91196, abcam, USA), anti-cTNT antibody from mouse (MS-295-P1, Thermo Fisher Scientific, USA), anti-cTNT antibody from rabbit (15513-1-AP, Proteintech, China), anti-α-actinin antibody (A7811, Sigma, USA), anti-SHOX2 antibody (ab55740, abcam, USA), anti-TBX18 antibody (ab115262, abcam, USA), anti-COUPTFII antibody (PP-H7147-00, R&D, USA), anti-MLC2V antibody (MABT180, Sigma, USA), and anti-TBX3 antibody (ab154828, abcam, USA), followed by the following species-specific fluorescence-conjugated secondary antibodies: alexa fluor 488 labeled goat anti-rabbit IgG (A-11008, Invitrogen, USA), alexa fluor 488 labeled goat anti-mouse IgG (A-11001, Invitrogen, USA), alexa fluor 594 labeled goat anti-rabbit IgG (R37177, Invitrogen, USA), and alexa fluor 594 labeled goat anti-mouse IgG (A-11005, Invitrogen, USA). The cells were then counterstained using 0.5 μg/ml of DAPI (4083, Cell Signaling Technology, USA) for 15 min at room temperature. After rinsing with PBS, the chambers were mounted and visualized under fluorescence microscopy (IX83, Olympus, Japan). Corresponding antibody isotype control, mouse IgG (ab205719, abcam, USA), rabbit IgG (ab205718, abcam, USA), mouse IgG (ab190369, abcam, USA), mouse IgG1 (#5415, Cell Signaling Technology, USA), and mouse IgG3 (#37988, CST, USA) were used.

### Flow cytometry

The induced cardiomyocytes in 12-well plate were digested with 0.25% trypsin with 0.5 mM EDTA into single cell suspension and washed with PBS. Cells were fixed with 4% formaldehyde for 10 min at room temperature and chilled on ice for 1 min. Permeabilization was performed by adding one tenth of ice-cold 100% methanol slowly to the pre-chilled cells and continued to incubate on ice for 30 min. Cells were then blocked with blocking buffer (0.5% BSA in PBS) for 10 min; incubated with the following primary antibodies: anti-OCT4 antibody (#2750, Cell Signaling Technology, USA), anti-NANOG antibody (#3580, Cell Signaling Technology, USA), anti-SSEA4 antibody (#4755, Cell Signaling Technology, USA), anti-TRA-1-60 antibody (#4746, Cell Signaling Technology, USA), anti-Ki67 antibody (ab15580, abcam, USA), anti-cTNT antibody (MS-295-P1, Thermo Fisher Scientific, USA), anti-NKX2-5 antibody (ab91196, abcam, USA), and anti-SHOX2 antibody (ab55740, abcam, USA) for 1 h at room temperature; then washed with PBS; and followed by incubation with the corresponding species-specific fluorescence-conjugated secondary antibodies: alexa fluor 488 labeled goat anti-mouse IgG (A-11029, Invitrogen, USA), alexa fluor 488 labeled goat anti-rabbit IgG (A-11034, Invitrogen, USA), alexa fluor 647 labeled goat anti-mouse IgG (A-21235, Invitrogen, USA), and alexa fluor 647 labeled goat anti-rabbit IgG (A-32733, Invitrogen, USA) for 30 min at room temperature. Cells were analyzed using a flow cytometry machine (651155, BD FACS Verse, BD Bioscience, USA) according to the manufacturer’s protocol.

### Action potential (AP) recording

AP recording was performed following El-Battrawy et al.’s protocol with some modifications [20]. Briefly, on 60 days after cardiomyocyte differentiation, induced cardiomyocytes were dissociated into single cell suspension by 30 min type I collagenase (2 mg/ml) followed by 3 min trypsin (0.25%) without EDTA. 1 × 10^4^ cells were seeded into a 3.5-cm dish containing a lysine-treated glass coverslip and incubated for 3 days. AP was recorded using the whole cell patch clamp electrophysiology method. Briefly, the adherent cells on the coverslip were placed in the recording chamber and perfused with bath solution containing 140 mM NaCl, 1 mM MgCl_2_, 5 mM KCl, 1.8 mM CaCl_2_, 5 mM 4-(2-hydroxyethyl)-1-piperazineethanesulfonic acid (HEPES), and 10 mM glucose (the pH was adjusted to 7.40, and the osmolality to 301 ± 3 mOsm, respectively). The patch pipettes were pulled from borosilicate glass capillaries (7-000-0650-LHC, Drummond, USA) by a horizontal puller (PC100, NARISHIGE, Japan) and had resistances of 1.5–3 MΩ. Pipette solution consisted of 110 mM K-gluconate, 20 mM KCl, 1 mM CaCl_2_, 1 mM MgCl_2_, 10 mM HEPES, 5 mM ethylene glycol tetraacetic acidpotassium chloride (EGTA-KOH), 5 mM ATP-Mg^2+^, and 5 mM Na-phosphocreatine. The pH was adjusted to 7.2 by KOH, and the osmolality to 290 ± 3 mOsm. A Multiclamp 700B amplifier was used to record APs, and data were analyzed using a custom software.

### Statistical analysis

Experimental data are presented as “mean ± SD” with at least three repeats. Comparisons between multiple groups were performed using one-way analysis of variance (one-way ANOVA), with *p* < 0.05 considered as statistically significant.

## Results

### Characterization of hiPSC

**Fig. 1:**
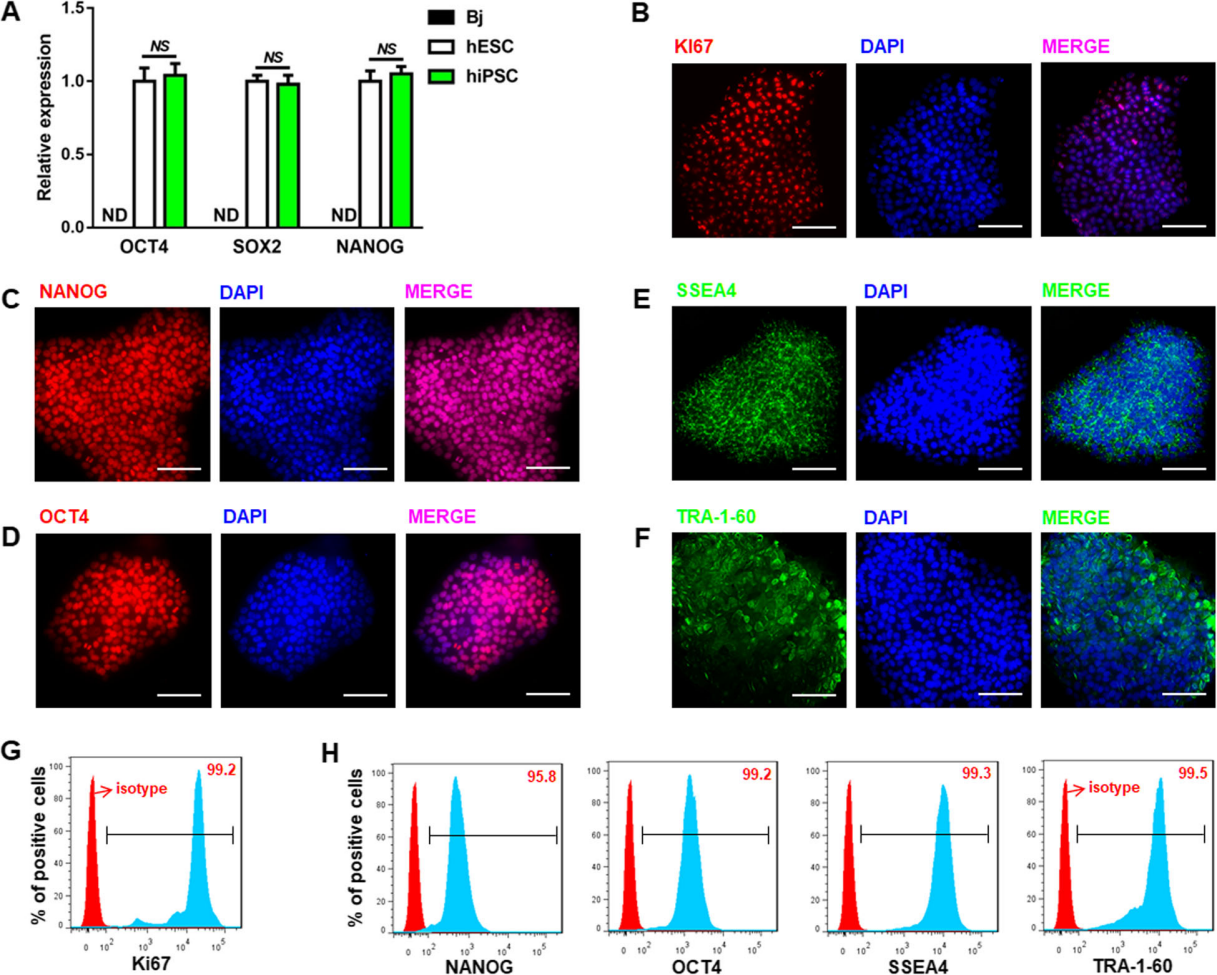
Characterization of the hiPSC. **a** hiPSC expressed high levels of the pluoripotency marker genes, OCT3/4, SOX2, and NANOG, which were comparable to hESC, a human embryonic stem cell (*t* test; NS, not significant; ND, not detectable; *n* = 3). **b** Ki67, pluoripotency markers of hiPSC were identified using IF method. **c**–**e** Pluoripotency markers of hiPSC (NANOG, OCT4, SSEA4, and TRA-1-60) were confirmed by immunoflorescence (IF) assay. **g**, **h** Representative flow cytometry analysis further confirmed the pluoripotency of the hiPSC. Scale bars, 100 μm (× 400). Expression values of all PCR analyses were normalized to the housekeeping gene GAPDH. Data are presented as “mean ± SD”

hiPSC and human embryonic stem cell (hESC) were purchased from OSINGLAY BIO company. To confirm the authenticity of the hiPSC, the molecular signature of hiPSC was validated by both qRT-PCR and immunofluorescence (IF). The qRT-PCR results showed that the hiPSC expressed stem cell-specific markers OCT3/4, SOX2, and NANOG at similar levels as hESC which serves as a positive control. In contrast, these markers were non-detectable in the terminal differentiated human terminal differentiated fibroblast cells (Bj) as a negative control (Fig. 1a). The IF results showed that the hiPSC was proliferative (Ki67 positive) (Fig. 1b) and heavily stained for NANOG, OCT4, SSEA4, and TRA-1-60, which were localized in the nuclei (for NANOG and OCT4) and plasma membrane (for SSEA4 and TRA-1-60), respectively (Fig. 1c–f). This was further confirmed using flow cytometry analysis (Fig. 1g, h). These results demonstrate that the hiPSC possesses their pluripotency and self-renewal capability.

### Identification of hiPSC-induced cardiomyocytes

**Fig. 2:**
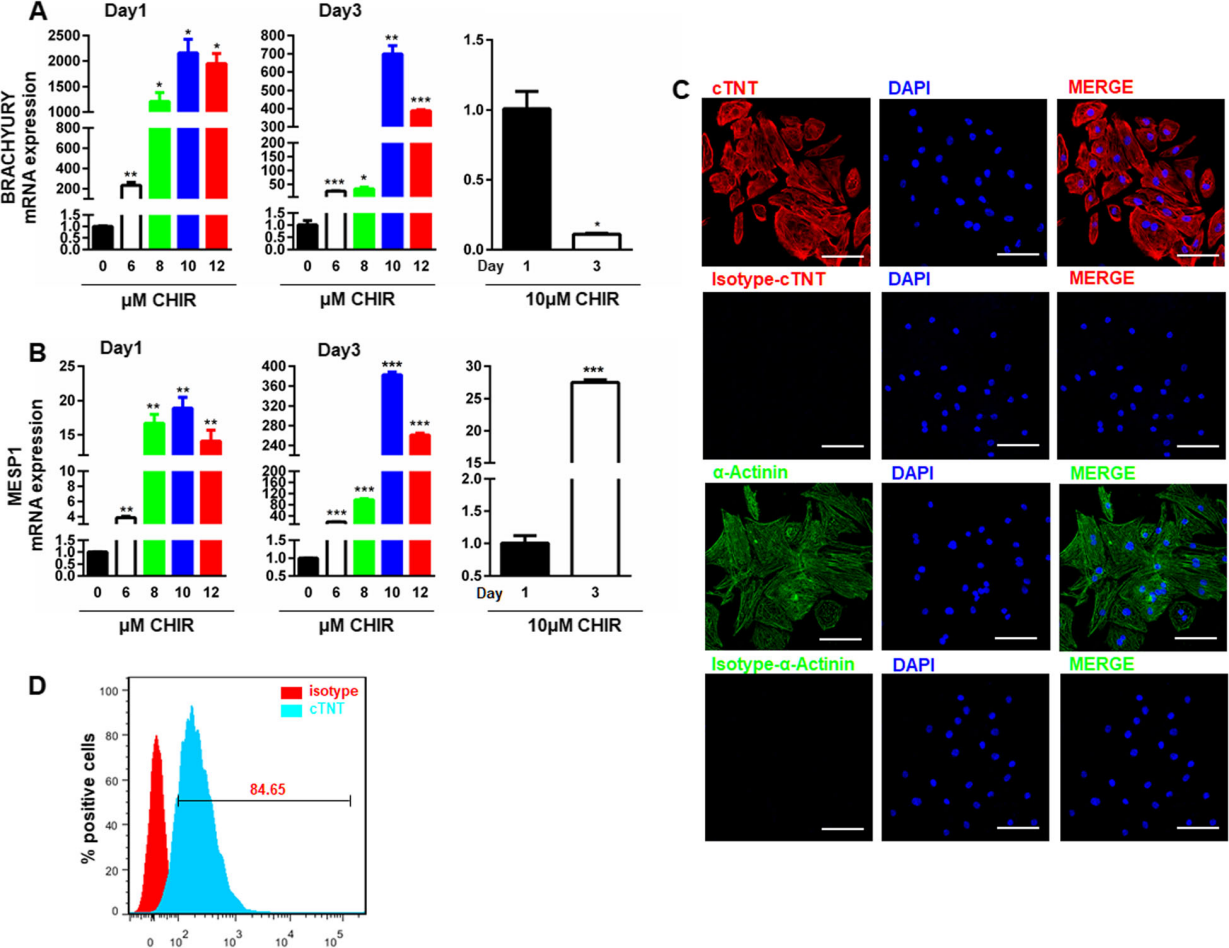
Characterization of the hiPSC-derived cardiomyocytes. **a**, **b** CHIR concentration-dependently increased the expression of BRACHYURY (**a**) and MESP1 (**b**) in differentiated cells at day 1 and day 3 (*t* test, *p < 0.05, **p < 0.01, and *** *p* < 0.001 versus 0 μM or day 1; *n* = 3). **c** The hiPSC-induced cardiomyocytes expressed cTNT and α-actinin indicated by IF assay in induced cardiomyocytes from day 21 differentiation. Scale bars, 100 μm (× 400). **d** Representative plots of flow cytometry displaying high yields of cardiomyocytes derived from hiPSC at day 21. Expression values of all PCR analyses were normalized to the housekeeping gene GAPDH. Data are presented as “mean ± SD’”

Cardiomyocytes were induced by the widely used GiWi protocol originally proposed by Lian et al. [19], where the induction process was temporally initiated using GSK3 inhibitor (Gi) and followed by temporal inhibition of the Wnt signaling pathway using Wnt inhibitor (Wi). To optimize the induction protocol, different concentrations of the Gi, CHIR were added on day 0 of the differentiation and continued to incubate for 24 h, and the markers of mesoendoderm (BRACHYURY) and cardiac mesoderm (MESP1) were evaluated on day 1 and day 3 using qRT-PCR. CHIR dose-dependently increased the expression of both BRACHYURY (Fig. 2a) and MESP1 (Fig. 2b) on day 1, with CHIR at 10 μM reached the highest level. Similar concentration-response curves were observed on day 3 for both markers (Fig. 2a, b). However, the overall levels of BRACHYURY (Fig. 2a) were significantly decreased, while the levels of MESP1 (Fig. 2b) were significantly increased, at different concentrations of CHIR compared to those levels on day 1. These observations suggest fate conversion from mesoendoderm into cardiac mesoderm. The successful induction of cardiomyocytes at day 21 from differentiation was confirmed by both IF, showing positive staining for cardiomyocyte markers, cTNT and α-actinin (Fig. 2c), and flow cytometry, showing that around 85% of the induced cardiomyocytes expressed the cardiac sarcomere proteins, cTNT (Fig. 2d). Quite high percent of beating cardiomyocytes was observed at day 21 as shown in SUPPL-Video 1. This is in sharp contrast with the spontaneously differentiated cells without GiWi treatment in SUPPL-Video 2. These observations further confirmed our optimized differentiation procedure.

### BMP, RA, and FGF signaling pathways contribute differently to the differentiation of cardiomyocytes toward SAN-like cells

**Fig. 3:**
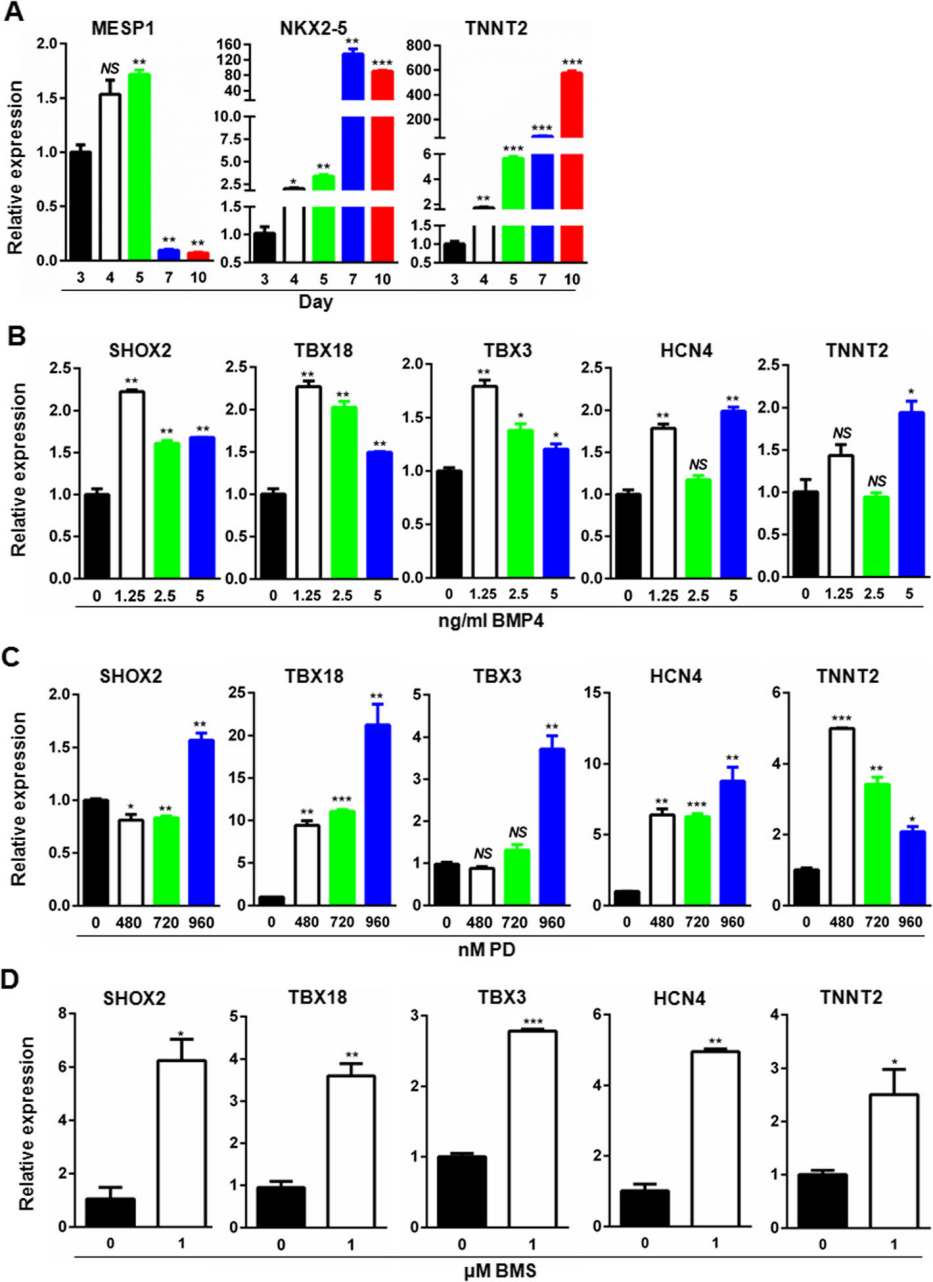
Optimization of the timing and concentration of small molecule chemicals targeting FGF, RA, and BMP signaling pathways for enriched SANLC differentiation. **a** The expression of cardiac mesoderm marker (MESP1), cardiac progenitor marker (NKX2-5), and cardiomyocyte marker (TNNT2) at days 3, 4, 5, 7, and 10, respectively, by qRT-PCR (*t* test, *p < 0.05, **p < 0.01, and *** *p* < 0.001 versus day 3; *n* = 3). **b** The expression of SHOX2, TBX18, TBX3, HCN4, and TNNT2 was evaluated by qRT-PCR at day 16 after the differentiation of hiPSC-induced cardiomyocytes was treated with BMP4 at the indicated concentrations at day 5–7 (*t* test, *p < 0.05, **p < 0.01, and NS, not significant versus 0 μM; *n* = 3). **c** The expression of SHOX2, TBX18, TBX3, HCN4, and TNNT2 was analyzed by qRT-PCR at day 16 of the differentiation after the induced cardiomyocytes were treated with PD at the indicated concentrations at day 5–7 (*t* test, *p < 0.05, **p < 0.01, ***p < 0.001, and NS, not significant versus 0 μM; *n* = 3).**d** The expression of SHOX2, TBX18, TBX3, HCN4, and TNNT2 was analyzed by qRT-PCR at day 16 of the differentiation after the induced cardiomyocytes were treated with BMS at the indicated concentrations at day 5–7 (*t* test, *p < 0.05 and **p < 0.01 versus 0 μM; *n* = 3). Expression values of all PCR analyses were normalized to the housekeeping gene GAPDH. Data are presented as “mean ± SD”

**Fig. 4:**
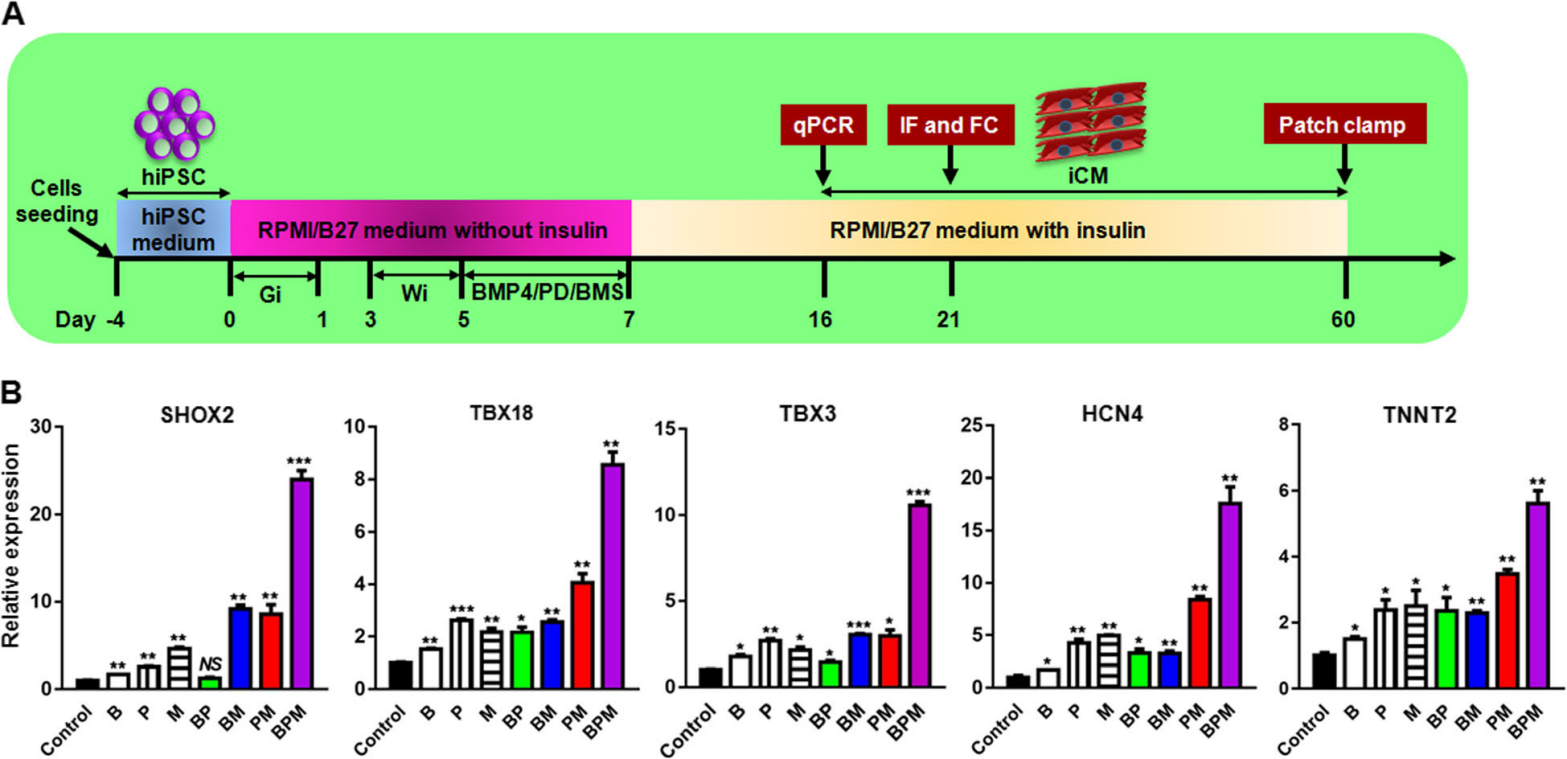
The BMP4/PD/BMS (BPM) method promotes the differentiation of hiPSC toward SANLC. **a** Scheme of the GiWi-based protocol used for enriched differentiation of SANLC from hPSC via treatment of BMP4, PD, and BMS. Gi, GSK inhibitor (CHIR); Wi, Wnt inhibitor (IWP2); iCM, induced cardiomyocytes. **b** Compared to the GiWi control, the levels of the expression of SHOX2, TBX18, TBX3, HCN4, and TNNT2 (from left to right) generally displayed the gradual increasing tendency from the individual treatment of BMP4 (B), PD (P), and BMS (M) to the two combination of BMP4 + PD (BP), BMP4 + BMS (BM), and PD + BMS (PM), and BMP4 + PD + BMS (BPM) caused the highest expression of the markers above as shown by qPCR analysis at day 16 (*t* test, *p < 0.05, **p < 0.01, ***p < 0.001, and NS, not significant versus GiWi control; *n* = 3). Expression values of all PCR analyses were normalized to the housekeeping gene GAPDH. Data are presented as “mean ± SD”

Although it is generally agreed that ventricular, atrial, and SAN-like cardiomyocytes are derived from different progenitor cells emerging as early as mesoderm stage, and different signaling pathways contribute differently to the directional differentiation toward ventricular, atrial, and SAN-like cardiomyocytes, the timing of manipulation of the signaling pathways remains controversial. To determine the timing of manipulation, we evaluated the temporal expression of cardiac mesoderm marker (MESP1), cardiac progenitor marker (NKX2-5), and pan-cardiomyoyte marker (TNNT2) by qRT-PCR. As shown in Fig. 3, with the progression of cardiomyocyte differentiation, the expression of MESP1 reached the highest level on day 5 and sharply dropped on days 7 and 10, suggesting that day 5 is equivalent to the developmental cardiac mesoderm stage. In contrast, NKX2-5 expression dramatically increased and reached the highest level on day 7 equivalent to the cardiac progenitor stage, and TNNT2 were upregulated in a time-dependent manner (Fig. 3a). These results suggest that it is from day 5 to day 7 during which cells experienced fate conversion from the cardiac mesoderm to the progenitor. Given that the SANLC development originates as early as cardiac mesoderm, the optimal time window of 48 h between day 5 and day 7 was determined to manipulate the SANLC-enriched differentiation process using different signaling pathway modulators. The schematic illustration of SANLC-enriched differentiation protocol based on the GiWi method was shown in Fig. 4a.

BMP4 has been shown to increase the proportion of SANLC from hiPSC via activating the bone morphogenetic protein (BMP) signaling pathway. To optimize the concentration of BMP4 on the differentiation of hiPSC to the SANLPC, hiPSC was stimulated with different concentrations of BMP4 for 2 days and the expression of SAN markers SHOX2, TBX18, TBX3, and HCN4 were analyzed at day 16. Consistent with a previous report that lower concentration of BMP4 could increase the proportion of SANLPC from hiPSC, BMP4 at 1.25 ng/ml maximumly increased the expression of the SAN markers, SHOX2, TBX18, TBX3, and HCN4, but not the pan-cardiomyocyte marker TNNT2 that showed no response at lower concentrations and reached statistical significance only at 5 ng/ml (Fig. 3b). Interestingly, further increased concentrations of BMP4 tended to concentration-dependently weaken the effect of BMP4 on the expression of TBX18, SHOX2, and TBX3 (Fig. 3b). 1.25 ng/ml per milliliter was therefore chosen as the optimal concentration for BPM4.

NKX2-5-mediated fibroblast growth factor (FGF) signaling has been proposed to contribute to the differentiation of hiPSC to ventricular cardiomyocytes [13, 14]. To optimize the inhibition of FGF signaling pathway on the SANLPC generation, the FGF inhibitor PD was added in the hiPSC and the expression of SHOX2, TBX18, TBX3, HCN4, and TNNT2 were analyzed at day 16. The result showed that PD concentration-dependently upregulated all the SANLPC markers. As to TNNT2, PD at 450 nM significantly upregulated its expression, which was inhibited by increasing concentrations of PD in a concentration-dependent manner (Fig. 3c). Taken together, these observations indicate that PD at concentration of 960 nM is optimal.

Finally, the effect of retinoid acid (RA) signaling on SANLPC differentiation was also investigated at day 16. Treatment of hiPSC with 1 μM of the RA signaling inhibitor BMS significantly increased the expression of the markers of pacemaker cells HOX2, TBX18, TBX3, and HCN4 and the pan-cardiomyocyte marker TNNT2 (Fig. 3d).

### Combined modulation of the three signaling pathways synergistically promoted the differentiation of hiPSC to SANLC

Having optimized the conditions of manipulating bone morphogenetic protein (BMP), fibroblast growth factor (FGF), and retinoid acid (RA) signaling pathways individually on the differentiation of hiPSC to SANLC, we tested whether combined usage of the three modulators BMP4, PD, and BMS (they are abbreviated as B, P, and M correspondingly in Fig. 4b) has any synergistical effect. The scheme of SANLC-enriched differentiation protocol based on GiWi method is shown in Fig. 4a, where the detailed information regarding time points for the treatment and testing analysis was included. As shown in Fig. 4b, BMP4 + PD, BMP4 + BMS, PD + BMS, and BMP4 + PD + BMS (they are abbreviated as BP, BM, PM, and BPM correspondingly in Fig. 4b) combinations all significantly increased the expression of the pacemaker cell markers SHOX2, TBX18, TBX3, and HCN4 and the pan-cardiomyocyte marker TNNT2 compared to those of the GiWi protocol. Among the four combinations, BPM had the best induction efficiency (Fig. 4).

### The BMP4 + PD + BMS (BPM) induction protocol leads to biased differentiation of hiPSC to SANLC

**Fig. 5:**
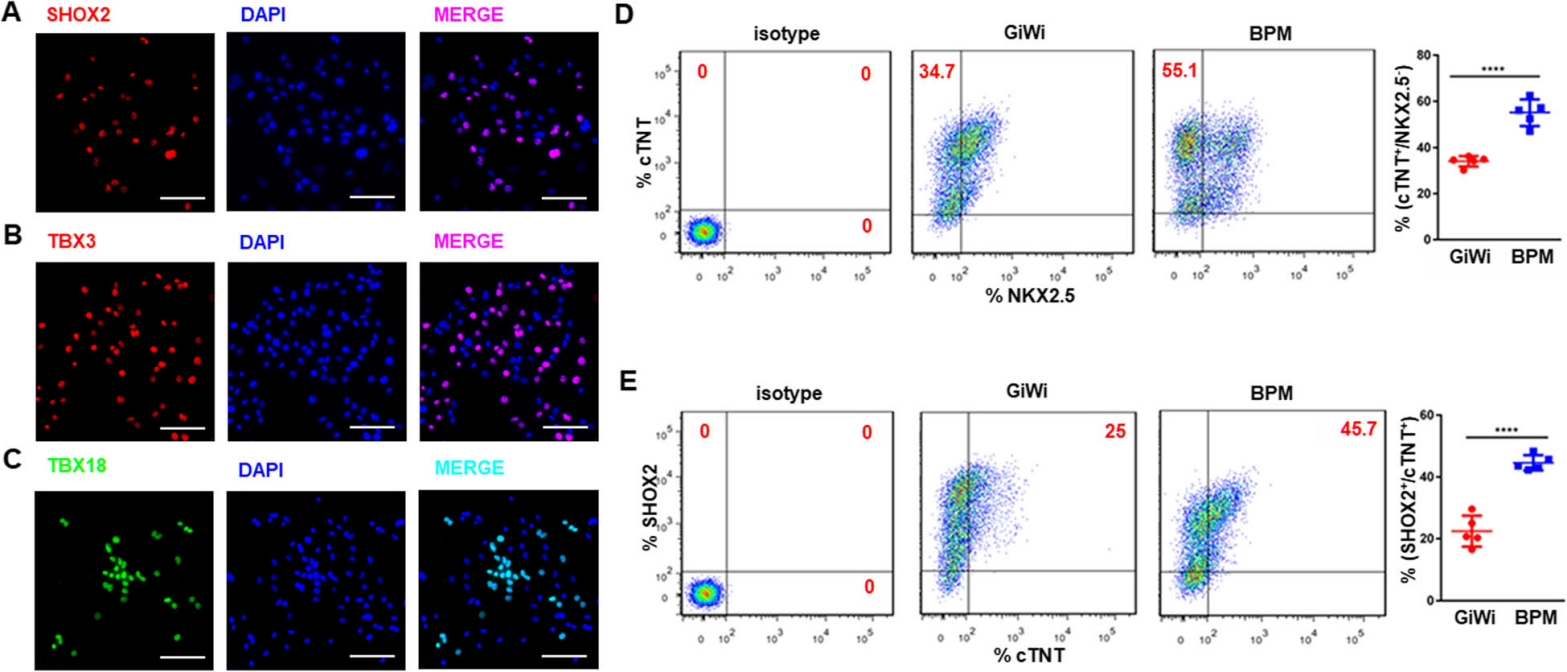
Validation of the enriched differentiation of SANLC by the BMP4/PD/BMS (BPM). **a**–**c** Representative IF analysis showed that SANLCs expressed SAN-specific transcription factor, SHOX2, TBX3, and TBX18. Scale bars, 100 μm (× 400). **d** Representative flow cytometry analysis showed that BPM significantly increased the percentage of CTNT^+^/NKX2.5^−^ cells as compared to the GiWi group (*t* test, ***p < 0.001 versus GiWi control; *n* = 5). **e** Representative flow cytometry analysis showed that BPM significantly increased the percentage of CTNT^+^/SHOX2^+^ cells as compared to the GiWi group (*t* test, ***p < 0.001 versus GiWi control; *n* = 5). Data are presented as “mean ± SD”

Since the BMP4 + PD + BMS (BPM) induction protocol most effectively increased the gene expression of the markers of pacemaker cells, we next evaluated the expression of the aforementioned markers at protein level using IF and flow cytometry on day 21. IF results showed that most nuclei were positive for transcription factors SHOX2, TBX3, and TBX18 (Fig. 5a–c). Likewise, flow cytometry data showed that BPM induction protocol generated a higher percentage of SANLC (defined by cTNT^+^/NKX2.5^−^) (55.1 ± 5%) compared to the traditional GiWi induction protocol (34.1 ± 2%) (Fig. 5d). When SANLC was defined by cTNT^+^/SHOX2^+^, the BPM protocol again generated a higher percentage of SANLC (44.5 ± 2% in the BPM versus 22.4 ± 5% in the GiWi) (Fig. 5e). The results of Fig. 5d, e were further confirmed by IF analysis of double staining for cTNT/NKX2.5 and cTNT/SHOX2 as shown in SUPPL-Fig. 1. It seemed that the SAN cells identified by cTNT^+^/NKX2.5^−^ and cTNT^+^/SHOX2^+^ were more likely to display the similar morphological characteristics indicated by elongated or spindle-like shapes that are consistent to the previous report.

It may be inferred that enriched SANLC population by BPM resulted in the reduced ratio of other two cardiomyocyte subtypes: ventricular and atrial cells. To further evaluate the effect of BPM, the expression of ventricular and atrial related markers was tested using qPCR analysis at day 16. Consistent with our hypothesis, the levels of both atrial (MYL7 and COUPTFII) (SUPPL-Fig. 2A) and ventricular (MYL2 and NKX2.5) (SUPPL-Fig. 2B) markers were significantly decreased in the BPM group. These results were further supported by IF analysis of COUPTFII and MLC2V whose coding gene is MYL2 (SUPPL-Fig. 2C). The markers of another automatic cell subtype atrioventricular node (AVN) cell were investigated using qPCR analysis at day 16. The results showed that the expression of SAN markers MSX2 and TBX2 was reduced by BPM (SUPPL-Fig. 3).

### SANLC generated by the BPM protocol displays the typical electrophysiological characteristics of pacemaker cells

**Fig. 6:**
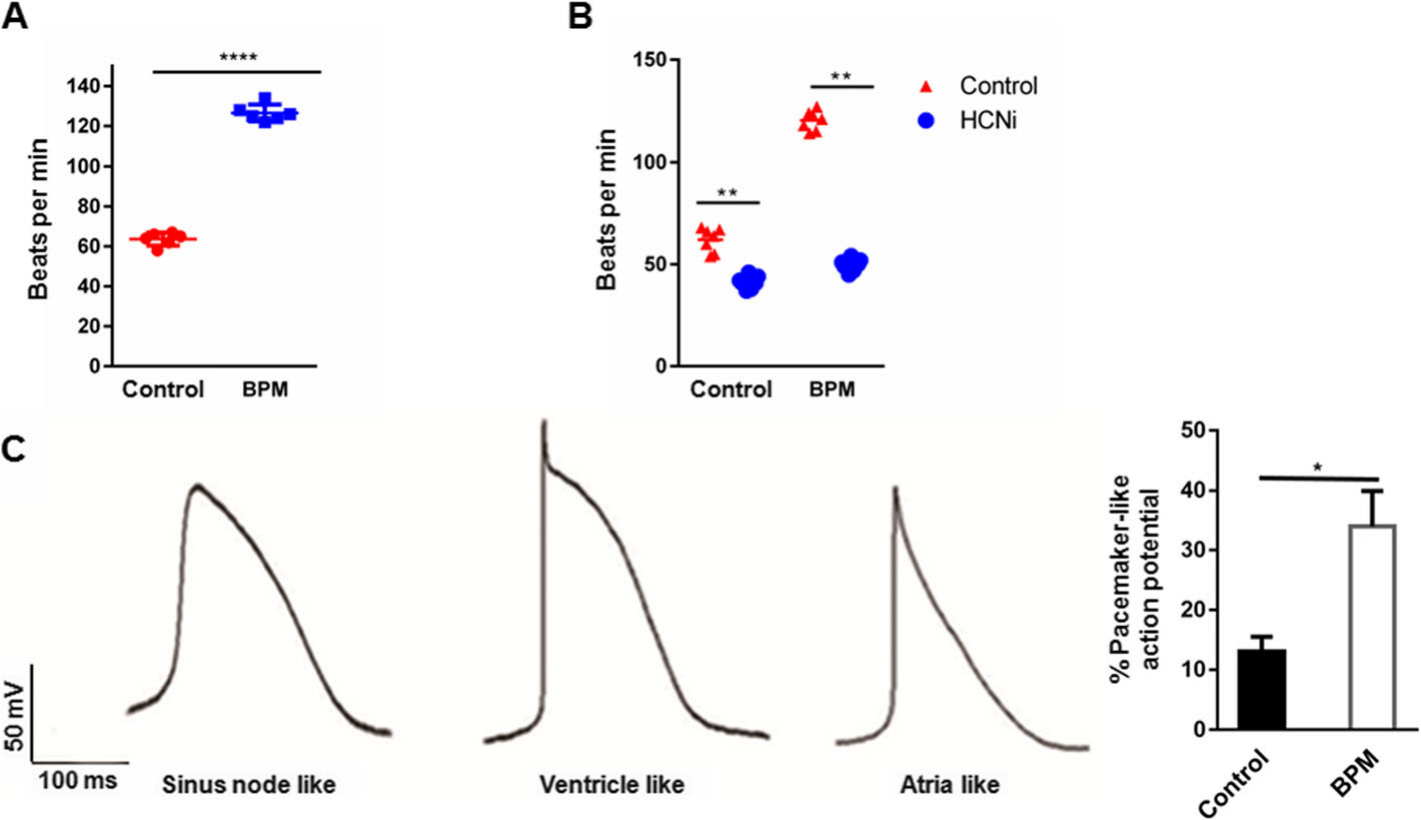
SANLCs induced by the BMP4/PD/BMS (BPM) possess typical electrophysiological characteristics of SAN. **a** Spontaneous beating frequency of SANLCs was significantly higher in the BPM (*n* = 6) than in the GiWi groups (*n* = 6) (*t* test, ***p < 0.001 versus GiWi control followed by Bonferroni’s post hoc test). **b** HCN4 channel inhibitor treatment caused significantly decreased beating rate in the BPM (*n* = 7) compared to the GiWi groups (*n* = 7) (*t* test, **p < 0.01 versus GiWi control followed by Bonferroni’s post hoc test). **c** Representative ventricule-like, atria-like, and sinus node-like action potential (AP) curves were recorded by whole cell patch clamp (left panel). The percentage of cells with pacemaker-like AP was remarkably increased in the BPM compared to the GiWi group (6/18 versus 2/15, BPM versus GiWi) (*t* test, ***p < 0.001 versus GiWi control). Data are presented as “mean ± SD”

To evaluate the automaticity of the SANLC, cells were observed under microscopy and the beating rate was recorded. The representative movies of beating cardiomyocytes shown in SUPPL-Video 3 (GiWi group) and SUPPL-Video 4 (BPM group) displayed that the beating rate of SANLC was significantly higher in the BPM protocol (*n* = 6) than in the GiWi protocol (*n* = 6) (Fig. 6a). Since hyperpolarization-activated cyclic nucleotide-gated channel (HCN4) contributes the most to the automaticity in SAN, inhibition of the HCN4 would decrease the automaticity of the SANLC. Indeed, inhibition of the HCN4 using zatebradine hydrochloride significantly decreased the beating rate of SANLC from the BPM protocol (*n* = 7) than the GiWi protocol (*n* = 6), suggesting a higher percentage of SANLC in the BPM protocol than in the GiWi protocol (Fig. 6b). We then analyzed the AP using whole cell patch clamp technique 60 days after the differentiation. Based on the morphology of AP, ratio of AP duration at 90% repolarization (APD90) to APD50, upstroke velocity, and maximum diastolic potential, SANLC from both groups contained ventricle-like, atrial-like, and pacemaker-like cells (Fig. 6c). However, the percentage of pacemaker-like cells was significantly higher in the BPM protocol than in the GiWi protocol (Fig. 6c).

Different expression profiles of the ion channels could account for the different electrophysiological properties among SAN, ventricular, and atrial cell subtypes. To further confirm the SANLC-biased effect of BPM, a series of ion channel genes were checked by qPCR at day 16. In addition to the SANLC markers (SHOX2, TBX18, TBX3, and HCN4) (Figs. 4 and 5), two other SANLC markers ISL1 and HCN1 displayed increased expression levels by BPM treatment (SUPPL-Fig. 4A). The qPCR analysis of potassium ion channels showed that BPM increased the SANLC-specific channel genes (KCND2, KCNK2, and KCNN4) but reduced the levels of ventricle specifically expressed gene KCNQ1 and atria specifically expressed gene KCNJ5 (SUPPL-Fig. 4B). Regarding the calcium ion channels, the expression of SANLC channel genes (CACNA1A and CACNB1) were upregulated by BPM, whereas a ventricular ion channel marker CACNA1C was reduced (SUPPL-Fig. 4C). Similar result of sodium ion channel was obtained, as evidenced by increased pacemaker cell specifically expressed gene SCN3B and decreased ventricle specifically expressed gene SCN5A (SUPPL-Fig. 4D). In addition, the result of testing the gap junction channel related genes showed that BPM treatment caused remarkable induction of SANLC-specific CX30.2. However, expression for both ventricular CX43 and atrial CX40 was decreased (SUPPL-Fig. 4D).

## Discussion

In this study, using the system of cardiomyocyte differentiation from hPSC by temporally manipulating the canonical Wnt signaling pathway for cardiac development stimulation, we discovered that SANLCs can be significantly enriched by simultaneous manipulation of BMP, FGF, and RA signaling pathways. These biasedly enriched SANLCs express SAN-specific markers that are sensitive to HCN4 channel blocker, and possess the electrophysiological property of native SAN cardiomyocytes.

Biased differentiation of cardiomyocytes to SANLCs could be achieved by either manipulating the expression of SAN-specific transcription factors or using pathway-specific activators/inhibitors. For example, TBX18 is restrictedly expressed in SAN where it promotes the development of pacemaker cardiomyocytes and at the same time prevents the activation of genes leading to chamber cardiomyocyte development. Accordingly, forced expression of TBX18 in hiPSC resulted in increased differentiation to SAN-like cardiomyocytes [21]. In addition, overexpression of TBX18 could convert the human working cardiomyocytes, adult rat bone mesenchymal stem cell, and adipose-derived stem cells into functional SAN-like cardiomyocytes [22, 23]. However, these methods of genetic manipulation for SAN regeneration are not desirable in the future clinical applications.

Previous investigations have demonstrated that SAN-like cells could be induced from different cell types by gene modification-dependent strategy. In our study, we established a gene-free and chemical-induced method for highly efficient differentiation of SAN-like cells from hiPSC, which may be more amendable in future clinical use. In a recent study, Protze et al. introduced a gene-free method for SAN cell induction from hPSC [24]. Based on the system of embryonic body-based cardiomyocyte differentiation, they showed that modulating the BMP and RA signaling pathway enables highly efficient NKX25^−^/cTNT^+^ SAN cell induction (55 ± 5%) indicated by flow cytometry results which is similar to our results (55.1 ± 5%). It was suggested that transgene-independent method may serve as a faster, simpler, and higher efficient strategy for SAN cell generation.

BMP signaling has been reported to participate in the induction of cardiac mesoderm and formation of the first heart field [17, 25]. Low concentration of recombinant BMP4 could induce cardiac mesoderm specification from hPSC and, more importantly, increase the proportion of SAN-like progenitor cells in a time window- and concentration-sensitive manner [8]. In line with these observations, our investigation showed that treatment of hiPSC with low concentration of BMP4 during the cardiac mesodermal stage increased the yields of SAN-like cardiomyocytes. However, by increasing the dosage of BMP4, it weakened the expression of SAN-specific markers.

FGF signaling is indispensable for promoting and maintaining the characteristics of ventricle during the early stage of heart development. Activation of FGF signaling sustains ventricular development in the early stage by maintaining the number and the electrical characteristics of ventricular cardiomyocytes, while inhibition of FGF signaling results in a gradual accumulation of atrial cardiomyocytes, and a concomitant decreasing number of ventricular cardiomyocytes [26]. Further studies showed that NKX2-5 is the downstream regulator of the FGF signaling [13, 14]. Indeed, our study found that inhibition of FGF signaling downregulated the expression of ventricle-specific makers including NKX25, while enhanced the expression of SAN makers.

RA signaling is not only essential for normal heart development but also involved in the differentiation and specification of atrial cardiomyocytes. Previous studies have shown that activation of RA signaling in hiPSC differentiation is sufficient to generate cardiomyocytes displaying both electrophysiological characteristics and gene expression profile of atrial cardiomyocytes. However, study by Protze et al. showed that activation of RA signaling increases the expression of some markers of SAN and does not affect the induction efficiency in hiPSC differentiation [24]. Our study showed that antagonizing RA signaling significantly increased the expression of SAN-specific markers SHOX2, TBX3, and HCN4 that promoted SAN differentiation. Considering the discovery by Protze et al. that timely activation of and the optimal activation of RA signaling promote the maturation of SAN, we tend to believe that the biological effect of RA signaling is not just involved in atrial development but also in SAN differentiation, which warrants future investigations. It is worth mentioning that the effect of RA in the SAN development may be in the spatiotemporal-dependent manner. In fact, Protze et al. showed that the effective time window of RA treatment enhancing pacemaker characteristics is 3–7 days in the hiPSC differentiation [24].

The effect of signal pathway modulators has been generally considered to be sensitive to the concentrations. For example, study by Protze et al. showed that BMP4 treatment at the low concentration range (1.25–5 ng/ml) could enhance the differentiation of SANLC from hiPSC [24]. In contrast, higher concentrations significantly inhibited the whole cardiomyocyte differentiation including SANLC [24]. Similarly, our study showed that the induction effect of BMP4 on the expression of SANLC markers gradually waned when increasing the concentrations (1.25, 2.5, 5 ng/ml) (Fig. 3b). One of the interesting observations is that the three combinations (BMP4 + PD + BMS) but not two combinations (BMP4 + PD, BMP4 + BMS, PD + BMS) demonstrated additional effect on the expression of SANLC markers when compared to the individual treatment (BMP4, PD, BMS). This suggests that the cardiomyocyte differentiation from hiPSC simulating the fetal heart development is a very complex process in a highly coordinated spatiotemporal manner, in which the terminal effect of combing different signaling pathways modulators was determined by a number of factors, such as concentration and time window.

### Study limitation

The present study has several limitations that are worth mentioning. A new efficient transgene-independent differentiation protocol for generating SANLC from hiPSC via combined modulating BMP, FGF, and RA signaling was not completely verified at the protein levels when compared to a broader coverage of the mRNA levels. However, several SANLC-specific markers (SHOX2, TBX18, and TBX3) in BPM-caused cells were confirmed by immunocytochemistry (Fig. 5a–c). The transcription factors coded by these three marker genes have generally been thought to play the most important role in SAN cell specification, patterning, and maturation [12, 24]. The mostly well-recognized double staining for cTNT/NKX2.5 was used to further verify the SANLC population induced by BPM treatment using flow cytometry and immunocytochemistry at the protein levles (Fig. 5d, SUPPL-Fig. 1) [24, 27], and it was further consolidated by another identification standard of SHOX2/cTNT (Fig. 5e, SUPPL-Fig. 1). Our in vitro studies demonstrated that BPM-enriched cells display the basic characteristics of SAN cells including higher beating rate, typical action potential, and sensitivity to the SAN cell-specific HCN4 channel inhibitor. However, the abilities of BPM-enriched cells to engraft within the heart and to drive the working cardiomyocytes at physiological rates in vivo animal model remain to be addressed. Our study showed that sequential modulation of Wnt signaling using the GiWi method was highly efficient enough to induce the pan-cardiomyocyte differentiation from hiPSC. However, we did not investigate the efficiency of Gi or Wi alone in consideration that Wnt signaling pathway has been shown to have biphasic effect on cardiac development in zebrafish, mouse embryos, and mouse embryonic stem cells with early Wnt signaling enhancing and later Wnt signaling repressing heart development [28, 29], suggesting that the cardiogenic effect of Wnt signaling pathway is highly timing sensitive and neither Gi nor Wi alone is not enough for highly efficient cardiomyocyte differentiation, and then high-efficiency SANCLs enrichment via combined modulation of MP, FGF, and RA signaling pathways based on the system of Gi or Wi alone.

## Conclusion

**Fig. 7:**
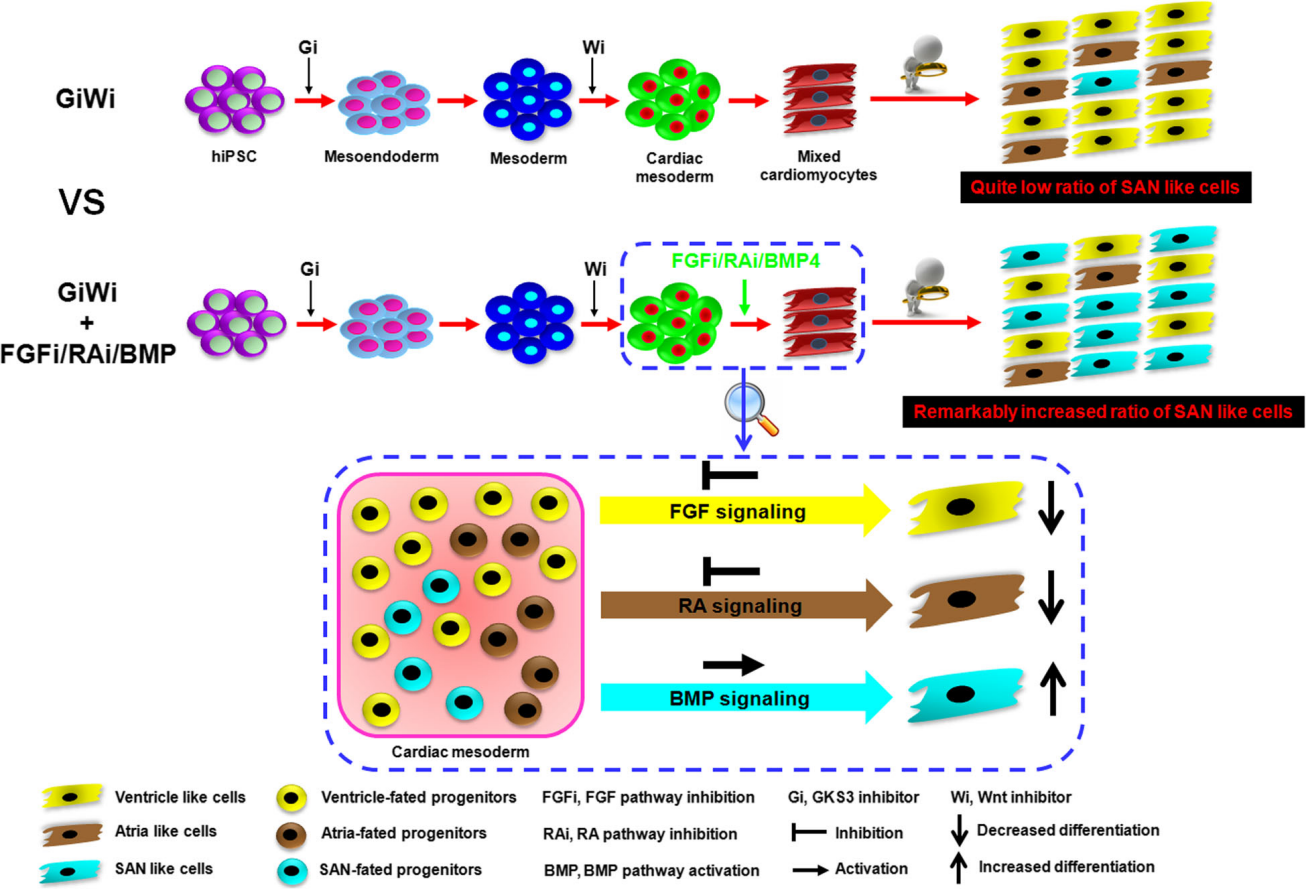
Graphic abstract. Working model of enriched differentiation of SANLC from hiPSC by the BMP4 + PD + BMS (BPM) treatment versus the GiWi method alone. Simultaneous activation of BMP and inhibition of RA and FGF signaling pathways during cardiac mesoderm stage of the GiWi-induced hiPSC differentiation strongly favor a SAN-specific gene transcriptional landscape leading to enhanced pacemaker cell specification

In summary, we used a developmental biology-guided approach to establish a transgene-independent highly efficient differentiation protocol for generating SAN-like cardiomyocytes from hPSC. We found that activation of BMP signaling and simultaneous inhibitions of both RA and FGF pathways during cardiac mesoderm stage of hiPSC differentiation lead to a SAN-specific gene expression landscape favoring pacemaker cell specification (Fig. 7). This provides a rich source of SAN cardiomyocytes to further study its biology and the potential applications in the treatment of arrhythmia-related disease.

## Supplementary information

**Additional file 1: Suppl-Fig. 1.** Validation of the enriched differentiation of SANLC by the BMP4/PD/BMS (BPM). Representative IF analysis at day 21 showed that both CTNT^+^/NKX2.5^−^ and CTNT^+^/SHOX2^+^ populations representing SANLC accounted for around 50% and 40% of the total cells indicated by DAPI respectively, which is consistent with the corresponding flow cytometry analysis. Scale bars, 100 μm (400×).

**Additional file 2: Suppl-Fig. 2.** Evaluation of the ventricular and atrial working cardiomyocytes in the BMP4/PD/BMS (BPM)-induced SANLC population. (A, B) Compared to the GiWi control, the expressions of specific atrial markers (MYL7 and COUPTFII) (A) and ventricle markers (MYL2 and NKX2.5) (B) were remarkably downregulated in BPM enriched SANLC at day 16 indicated by qPCR analysis (t test, * *p* < 0.05 versus GiWi control, *n* = 3). (C) Representative IF analysis further showed that quite low fractions of COUPTFII^+^and MLC2V^+^ (corresponding MYL2 gene) population was observed in total cells indicated by DAPI. Scale bars, 100 μm (400×). Expression values of all PCR analyses were normalized to the housekeeping gene GAPDH. Data are presented as ‘Mean ± SD’.

**Additional file 3: Suppl-Fig. 3.** Evaluation of the atrioventricular node (AVN) cells in the BMP4/PD/BMS (BPM)-induced SANLC population. Compared to the GiWi control, the expressions of specific AVN cells specific markers (MSX2 and TBX2) were significantly decreased in BPM group at day 16 as shown by qPCR analysis (t test, * p < 0.05 versus GiWi control, n = 3). Expression values of the PCR analysis was normalized to the housekeeping gene GAPDH. Data are presented as ‘Mean ± SD’.

**Additional file 4: Suppl-Fig. 4.** Evaluation of the expression of ion channel coding genes in the BMP4/PD/BMS (BPM)-induced SANLC population. A series of more other SANLC markers were checked using qPCR analysis 16 days after cell culture. (A) ISL1 (specific SANLC transcription factor) and HCN4 (SANLC specific pacemaker channel) were dramatically increased. (B-D) The expression of potassium, calcium and sodium ion channels distinguishing three types of cardiomyocytes (SANLC, ventricle and atria) were determined. (B) BMP treatment resulted in significant upregulation of SANLC potassium channel genes (KCND2, KCNK2 and KCNN4) in contrast with obvious decrease of ventricle (KCNQ1) and atria channels (KCNJ5). (C) Calcium ion channels related genes (CACNAIA and CACNB1) were increased by BPM while CACNA1C for ventricle was reduced. (D) BPM treatment remarkably increased the expression of SCN3B, SANLC specific sodium channel and decreased the level of SCN5, ventricle sodium channel. (E) Gap junction channel genes specific for SANLC (CX30.2), ventricle (CX43) and atria (CX40) were also tested. (t test, * p < 0.05 and ** *p* < 0.01 versus GiWi control, n = 3). Expression values of all PCR analysis was normalized to the housekeeping gene GAPDH. Data are presented as ‘Mean ± SD’.

**Additional file 5: Suppl Video 1**

**Additional file 6: Suppl Video 2**

**Additional file 7: Suppl Video 3**

**Additional file 8: Suppl Video 4**

## Data Availability

The datasets used and/or analyzed during the current study are available from the corresponding author on reasonable request.

## Abbreviations

PSC: Pluripotent stem cell
hiPSC: Human induced pluripotent stem cell
SA: Sinoatrial
SANLC: Sinoatrial node-like cell
SSS: Sick sinus syndrome
AVN: Atrioventricular node
BMP: Bone morphogenetic protein
FGF: Fibroblast growth factor
RA: Retinoic acid
IF: Immunofluorescence
AP: Action potential
GiWi: GSK3 inhibitor and Wnt inhibitor
