# Supplementary figures and images for: Enrichment differentiation of human induced pluripotent stem cells into sinoatrial node-like cells by combined modulation of BMP, FGF, and RA signaling pathways
Source: Stem Cell Res Ther. 2020 Jul 16;11:284. doi: 10.1186/s13287-020-01794-5 (PMC7364513; doi:10.1186/s13287-020-01794-5)

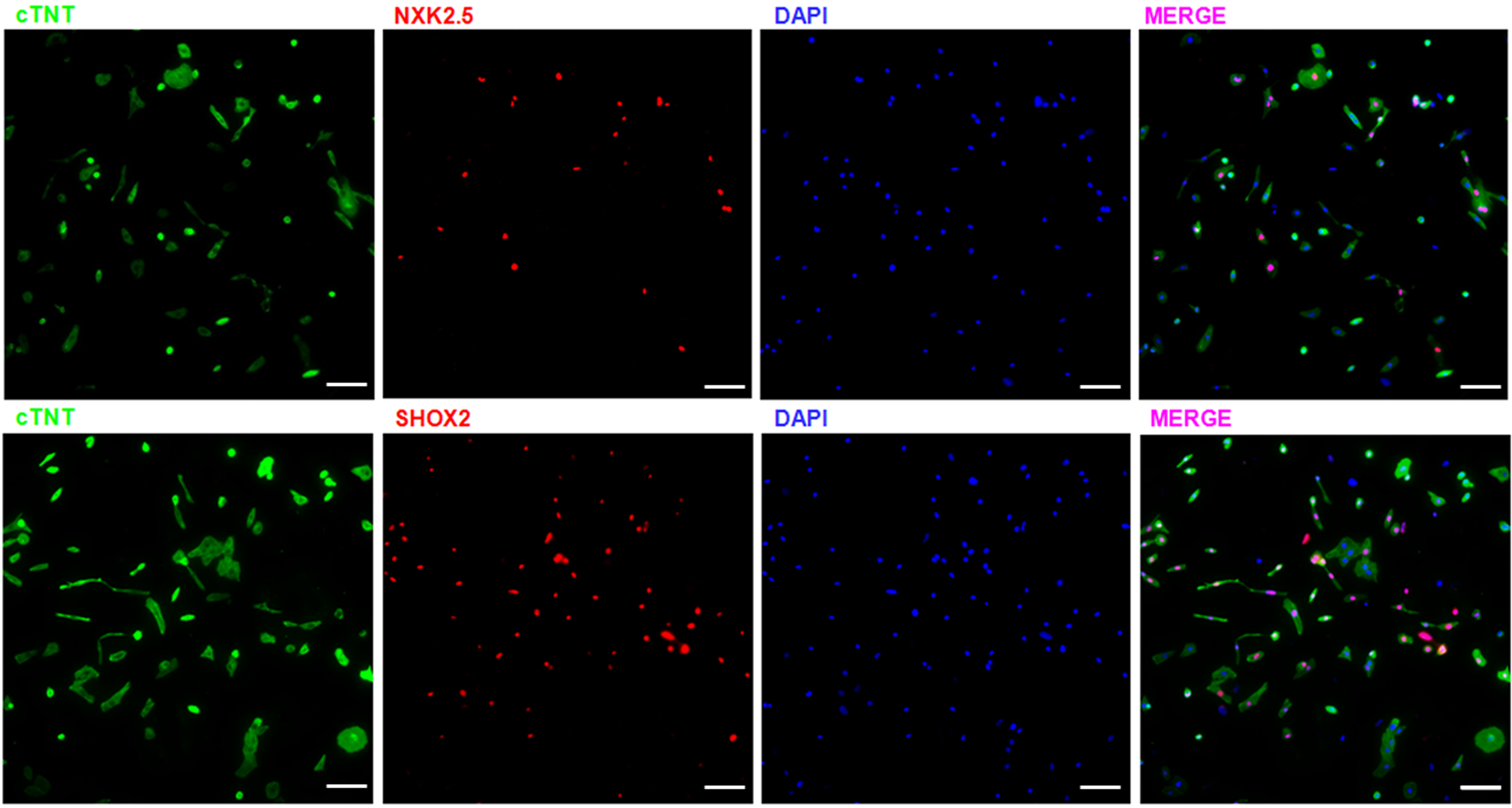

Supplement: Supplementary file 1 — Additional file 1: Suppl-Fig. 1. Validation of the enriched differentiation of SANLC by the BMP4/PD/BMS (BPM). Representative IF analysis at day 21 showed that both CTNT+/NKX2.5− and CTNT+/SHOX2+ populations representing SANLC accounted for around 50% and 40% of the total cells indicated by DAPI respectively, which is consistent with the corresponding flow cytometry analysis. Scale bars, 100 μm (400×). [file 13287_2020_1794_MOESM1_ESM.tif]

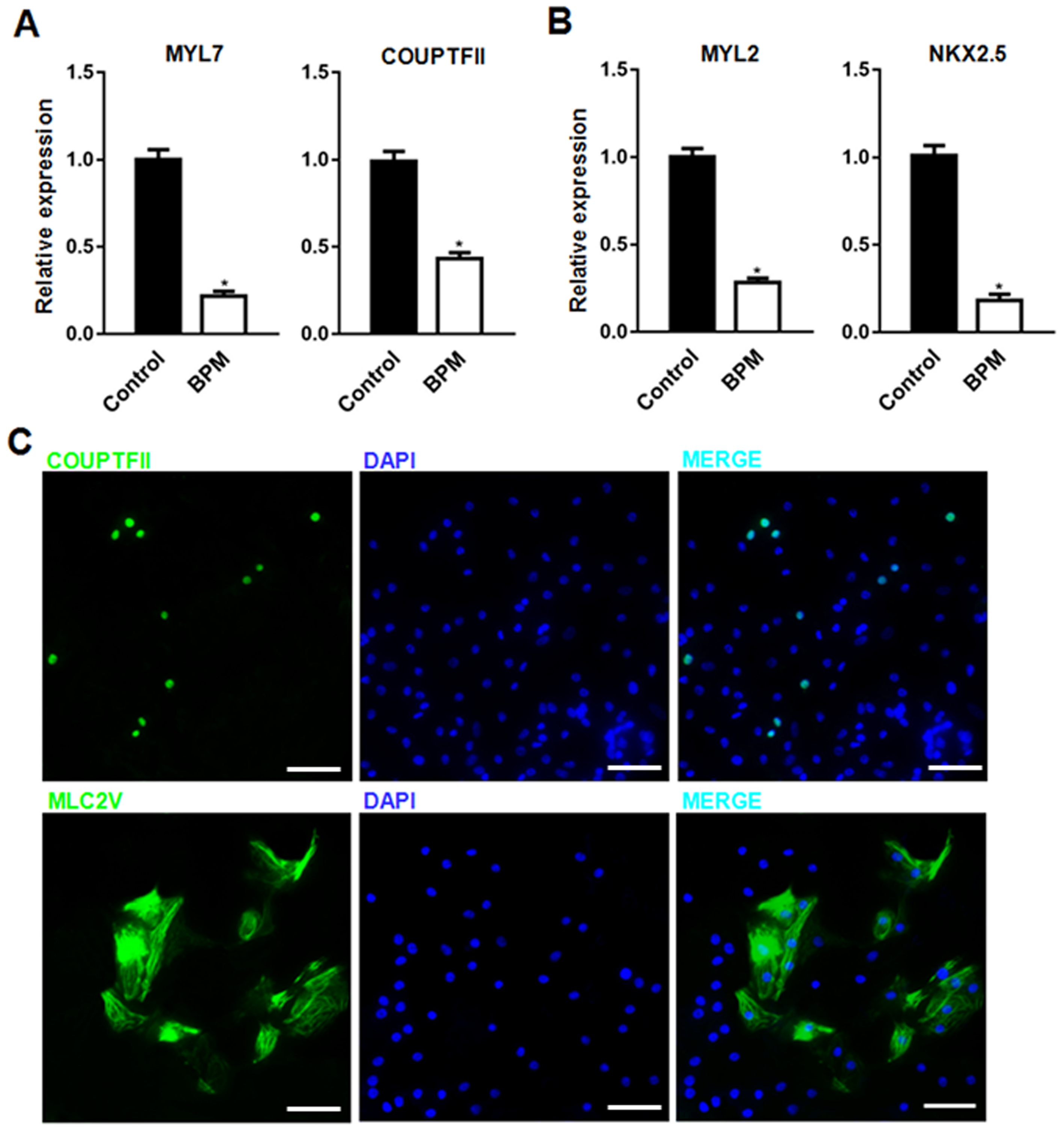

Supplement: Supplementary file 2 — Additional file 2: Suppl-Fig. 2. Evaluation of the ventricular and atrial working cardiomyocytes in the BMP4/PD/BMS (BPM)-induced SANLC population. (A, B) Compared to the GiWi control, the expressions of specific atrial markers (MYL7 and COUPTFII) (A) and ventricle markers (MYL2 and NKX2.5) (B) were remarkably downregulated in BPM enriched SANLC at day 16 indicated by qPCR analysis (t test, * p < 0.05 versus GiWi control, n = 3). (C) Representative IF analysis further showed that quite low fractions of COUPTFII+and MLC2V+ (corresponding MYL2 gene) population was observed in total cells indicated by DAPI. Scale bars, 100 μm (400×). Expression values of all PCR analyses were normalized to the housekeeping gene GAPDH. Data are presented as ‘Mean ± SD’. [file 13287_2020_1794_MOESM2_ESM.tif]

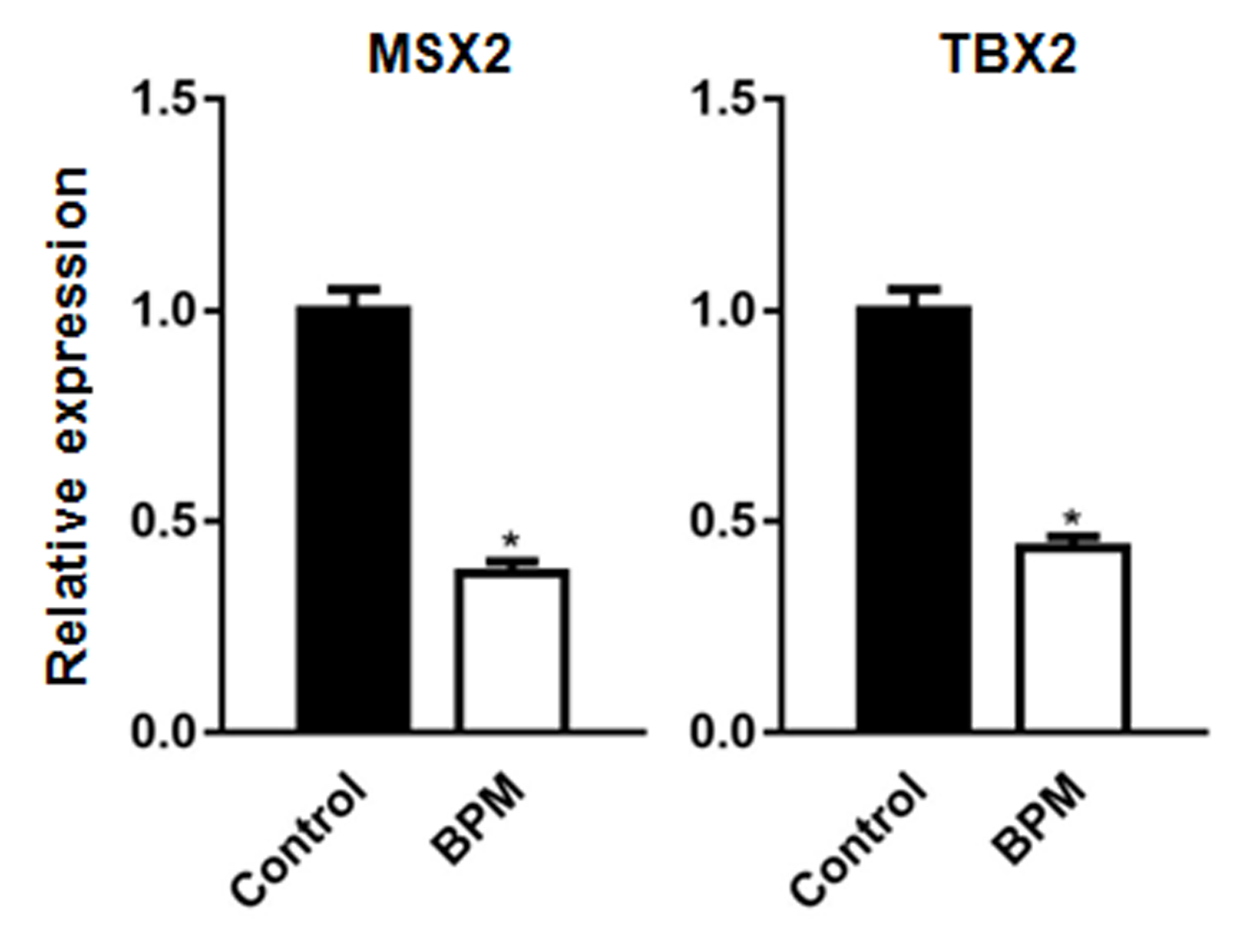

Supplement: Supplementary file 3 — Additional file 3: Suppl-Fig. 3. Evaluation of the atrioventricular node (AVN) cells in the BMP4/PD/BMS (BPM)-induced SANLC population. Compared to the GiWi control, the expressions of specific AVN cells specific markers (MSX2 and TBX2) were significantly decreased in BPM group at day 16 as shown by qPCR analysis (t test, * p < 0.05 versus GiWi control, n = 3). Expression values of the PCR analysis was normalized to the housekeeping gene GAPDH. Data are presented as ‘Mean ± SD’. [file 13287_2020_1794_MOESM3_ESM.tif]

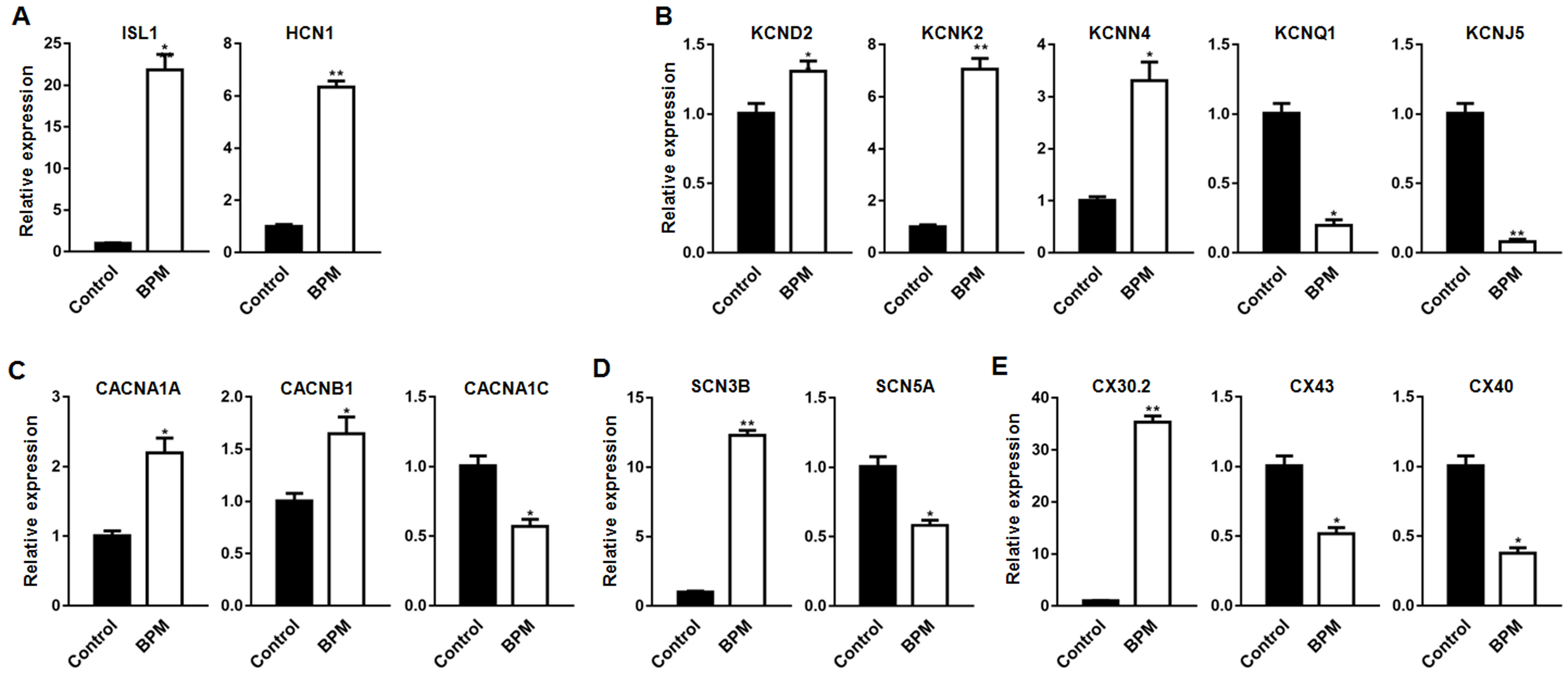

Supplement: Supplementary file 4 — Additional file 4: Suppl-Fig. 4. Evaluation of the expression of ion channel coding genes in the BMP4/PD/BMS (BPM)-induced SANLC population. A series of more other SANLC markers were checked using qPCR analysis 16 days after cell culture. (A) ISL1 (specific SANLC transcription factor) and HCN4 (SANLC specific pacemaker channel) were dramatically increased. (B-D) The expression of potassium, calcium and sodium ion channels distinguishing three types of cardiomyocytes (SANLC, ventricle and atria) were determined. (B) BMP treatment resulted in significant upregulation of SANLC potassium channel genes (KCND2, KCNK2 and KCNN4) in contrast with obvious decrease of ventricle (KCNQ1) and atria channels (KCNJ5). (C) Calcium ion channels related genes (CACNAIA and CACNB1) were increased by BPM while CACNA1C for ventricle was reduced. (D) BPM treatment remarkably increased the expression of SCN3B, SANLC specific sodium channel and decreased the level of SCN5, ventricle sodium channel. (E) Gap junction channel genes specific for SANLC (CX30.2), ventricle (CX43) and atria (CX40) were also tested. (t test, * p < 0.05 and ** p < 0.01 versus GiWi control, n = 3). Expression values of all PCR analysis was normalized to the housekeeping gene GAPDH. Data are presented as ‘Mean ± SD’. [file 13287_2020_1794_MOESM4_ESM.tif]
